# Farm-scale differentiation of active microbial colonizers

**DOI:** 10.1038/s43705-022-00120-9

**Published:** 2022-04-20

**Authors:** William L. King, Laura M. Kaminsky, Sarah C. Richards, Brosi A. Bradley, Jason P. Kaye, Terrence H. Bell

**Affiliations:** 1 Department of Plant Pathology and Environmental Microbiology, The Pennsylvania State University, University Park, PA 16802 USA; 2 Intercollege Graduate Degree Program in Ecology, The Pennsylvania State University, University Park, PA 16802 USA; 3 Intercollege Graduate Degree Program in International Agriculture and Development, The Pennsylvania State University, University Park, PA 16802 USA; 4 Department of Ecosystem Science and Management, The Pennsylvania State University, University Park, PA 16802 USA

**Keywords:** Microbial ecology, Soil microbiology, Applied microbiology

## Abstract

Microbial movement is important for replenishing lost soil microbial biodiversity and driving plant root colonization, particularly in managed agricultural soils, where microbial diversity and composition can be disrupted. Despite abundant survey-type microbiome data in soils, which are obscured by legacy DNA and microbial dormancy, we do not know how active microbial pools are shaped by local soil properties, agricultural management, and at differing spatial scales. To determine how active microbial colonizers are shaped by spatial scale and environmental conditions, we deployed microbial traps (i.e. sterile soil enclosed by small pore membranes) containing two distinct soil types (forest; agricultural), in three neighboring locations, assessing colonization through 16S rRNA gene and fungal ITS amplicon sequencing. Location had a greater impact on fungal colonizers (*R*^2^ = 0.31 vs. 0.26), while the soil type within the microbial traps influenced bacterial colonizers more (*R*^2^ = 0.09 vs. 0.02). Bacterial colonizers showed greater colonization consistency (within-group similarity) among replicate communities. Relative to bacterial colonizers, fungal colonizers shared a greater compositional overlap to sequences from the surrounding local bulk soil (*R*^2^ = 0.08 vs. 0.29), suggesting that these groups respond to distinct environmental constraints and that their in-field management may differ. Understanding how environmental constraints and spatial scales impact microbial recolonization dynamics and community assembly are essential for identifying how soil management can be used to shape agricultural microbiomes.

## Introduction

Soil microorganisms mediate many key ecosystem services, including global biogeochemical cycling. Although many soil microbial taxa can be observed using high-throughput sequencing, it remains challenging to distinguish between active microbes, dormant microbes, and relic DNA. Up to 95% of cells present in a given soil sample may be dormant [1–4], while up to 40% of characterized microbial richness may actually represent relic DNA [5]. At any point in time, the active portion of the microbiome is what contributes functionally to the environment, while the portion that is capable of active dispersal (i.e. potential dispersing pool) includes microbes that can place a high introduction effort on nearby soils and plant roots.

Microbial dispersal can enhance and/or restore local metabolic diversity and biomass, and has important roles in plant root colonization [2, 6, 7]. Although many studies show that soil microbiome composition is constrained by edaphic factors such as pH and organic matter [8–10], comparatively little is known about how composition is shaped by microbial dispersal [11]. Some biogeographical patterns in microbial composition have been observed [8, 9, 12, 13] and taxa are known to vary in their dispersal range [12, 14–16]. Even transient microbes, which do not persist long-term within microbiomes, can have important impacts on system function [17, 18]. Thus, approaches that can pinpoint microbes that move into a system within a specified time period would allow us to identify microbes that are potential near-term contributors to system function. Such an approach would also allow us to determine the scale at which the environment shapes the active microbial pool.

Agricultural settings offer a compelling system for studying microbial colonization and community assembly dynamics. The use of conventional management practices in agriculture has created unique microbial ecosystems, in some cases resulting in reduced microbial diversity, a depletion of key functional taxa, and weakened mutualistic plant-microbe interactions [19–23]. In particular, tillage is a commonly used soil management practice that disrupts soil microbial composition, especially existing fungal mycelial networks [24, 25]. For example, characterizing local sources of microbial propagules can be important for understanding which microorganisms are available to colonize and the successional dynamics following tillage. Because land use and quality can vary dramatically in agricultural systems, particularly when small farms are interspersed with less managed lands, there are unique opportunities to study microbial movement in neighboring habitats, including between-habitat microbial spillover [26]. Efforts to alleviate biodiversity loss in agricultural systems can include passive management practices, such as farmscaping and intercropping, which may also have impacts on soil microbial pools. The introduction of greater crop diversity or retaining patches of forest/gardens is thought to influence soil microbial diversity by stimulating microbial pools [27] and providing a source of microorganisms for passive dispersal into agricultural settings. However, it is not known whether or how these management practices have substantial influence on active microbial pools at farm-relevant spatial scales, or if instead microbial influx is overwhelmingly driven by broader forces like climate.

Bacteria and fungi likely differ in their ability to colonize new environments. Some fungi are known to be dispersal-limited at field scales [15, 28] and their distribution can be patchy [29], suggesting that the available pool of fungal colonizers could vary across similar and closely co-located soils. Several studies have shown the importance of priority effects in the development of fungal composition within a community (e.g. [30, 31]. Bacterial composition is thought to be more closely tied to soil physicochemical traits than fungal composition, in general [32], so at a farm scale, we would expect higher convergence of bacterial composition in soil patches with similar characteristics. In addition, we expect broader factors shaping microbial movement, such as precipitation, should disproportionately impact smaller organisms. Previous observations suggest that aerial deposition rates of viruses are magnitudes higher than rates of bacterial deposition [33], while aerosolized and deposited fungi appear to be magnitudes lower still [34–36]. Finally, we expect that the ability to create connected hyphal networks would give some fungi a greater ability to explore new and even sub-optimal environments than is possible for most bacteria. As a result, we would expect a disproportionate influence of the dominant fungi from a soil patch on the colonization of nearby roots and soil.

In this study, we aimed to contrast the recolonization and community assembly dynamics of active colonizing pools of bacteria and fungi and how they are shaped at farm-relevant spatial scales. Essentially, if within-farm location has substantial impacts on active microbial pools, then within-farmland management may have important roles in microbial management. Conversely, broad scale microbial dispersal (e.g. wind; rain) may overwhelm farm-scale management decisions. We used two sterile trap soils with different physicochemical characteristics, which we deployed across multiple transects that spanned three neighboring locations (farmland; managed grass strip; contiguous forest), in order to examine within- and between-location heterogeneity in microbial colonization at two timepoints. We hypothesized that: (i) each deployment location would have a unique microbial colonizing pool due to differing microbial dispersal influx among locations, (ii) bacterial colonizers, relative to fungal colonizers, would have greater compositional overlap with microorganisms found in the adjacent bulk soil microbiome, (iii) the physiochemical properties of the deployed trap soils would have a greater influence in structuring bacterial colonizer composition relative to fungal colonizer composition, and (iv) bacterial colonizers would have a greater degree of similarity between recolonized soils when comparing across locations relative to fungal colonizers. By understanding and contrasting patterns of bacterial and fungal colonization, we provide insights into the recolonization capacity and community assembly dynamics of microbial pools between neighboring locations and how the environment can structure active microbial pools at farm-relevant spatial scales.

## Methods

### Soil collection and preparation

Forest soil was collected from a Pennsylvania State University managed portion of the Rothrock State Forest (40° 42′ 45.9″ N 77° 55′ 53.4″ W), while the Farm (agricultural) soil was collected from an organic certified research farm (40° 43′ 17.4″ N 77° 55′ 34.9″ W), both at the Russell E. Larson Agricultural Research Center, managed by the Pennsylvania State University. For the forest soil, the organic matter layer was moved aside before soil collection. Both soils were collected from the A horizon at a depth of ~5–20 cm. Each collected soil was sieved through a 2.0 mm wire mesh sieve and mixed with sand (Quikrete medium sand; particle size 0.8–0.3 mm) to facilitate drainage, for a final sand content of 20%. Sieved soil was sterilized by autoclaving three individual times with 24 h between each autoclaving [37, 38]. Soil before and after sterilizing was analyzed by the Agricultural Analytical Services Laboratory at Pennsylvania State University (Supplementary Table 1). After the addition of sand and sterilization, the Sterile Forest soil was identified as a sandy loam textural class (sand: 56.7%; silt: 26.9%; clay: 16.5%), while the Sterile Farm soil was identified as loam (sand: 49.1%; silt: 29.4%; clay: 25.1%). Relative to the Sterile Farm soil, the Sterile Forest soil was characterized by a lower pH (5.6 vs. 6.7), and higher total nitrogen (0.72 vs. 0.09 %), total carbon (7.01 vs. 0.92%) and organic matter content (8.4 vs. 1.8%).

### Microbial trap development

Microbial traps were constructed with 24-well no-bottom plates (Greiner Bio-One, catalog: 662000-06) and a 18 µm nylon membrane (Tisch Scientific, catalog: ME17341, laser cut to 130 mm length and 90 mm height). The 18 µm pore size was chosen to allow microbial movement into the traps (e.g. bacterial cells and fungal hyphae), as in Albright and Martiny [11], and to restrict the entry of larger organisms such as protists. The 18 µm nylon membrane was attached to both sides of the 24-well no-bottom plates using DAP Silicone Max. The 24-well no-bottom plates, 18 µm nylon membranes, and all other trap construction equipment were immersed in absolute ethanol for 4–10 min and allowed to air dry in a UV-sterilized biological safety cabinet (BSC) prior to use. Sterilized soil from each treatment (forest or farm) was used to fill eight wells within the 24-well no-bottom plate, on opposite sides of the plate. Opposite sides of the plate were chosen to prevent cross-contamination of each soil during the assembly process.

### Microbial trap deployment

We deployed the microbial traps along three transects (referred to as A, B, and C; Supplementary Table 2) at the Russell E. Larson Agricultural Research Center (40° 42′ 53.0″ N 77° 55′ 51.2″ W) on 27 August 2020. Each transect had three separate deployment locations: (1) a conventionally managed agricultural (corn) farm (henceforth referred to as Farm), (2) neighboring contiguous forest, and (3) a grassy intermediary site (Supplementary Table 2). Transects were placed 20 m apart and deployment sites within a transect were 20 m apart. Microbial traps were embedded approximately 5–7 cm below the soil surface, in order to capture microbial colonizers in an active soil layer where most root activity occurs and to reduce noise from above-soil processes. At each deployment site, bulk soil was collected for soil analysis (Supplementary Table 1) by using an ethanol-washed garden trowel from same spot where we embedded the microbial traps. When deployed, microbial traps were watered with sterilized tap water and then covered with soil. Rain was also observed the following two days, with 4.1 and 30 mm of rainfall, respectively, ensuring adequate water saturation to facilitate in-soil microbial dispersal (weather station: USC00368449). In addition to the transects, an additional 18 µm microbial trap was deployed into an organically managed research farm (henceforth referred to as CCC plots) as a reference plot, also within the Russell E. Larson Agricultural Research Center (Supplementary Table 2). No active farm management occurred during the microbial trap incubation period.

### Microbial trap collection and DNA extraction

Microbial traps and the bulk soil directly underneath the traps were collected at two time intervals: one week (8 days) and ten weeks. The earlier timepoint was to examine immediate colonization by quick dispersers, while the later timepoint served to capture slower dispersers and subsequent colonization. An example of a fully constructed microbial trap following field-collection is provided as Supplementary Fig. 1. Once collected, microbial traps were immediately transported to the laboratory and stored at −20 °C until processed. Soil was collected from microbial traps within a UV-sterilized BSC. DNA was extracted from four replicates (i.e. four individual wells) of each soil from each microbial trap, and from the collected bulk soils in triplicate from each site and timepoint, using a NucleoSpin 96 Soil DNA extraction kit (Machery-Nagel; catalog: 740787.2) as per the manufacturer’s instructions. Microbial trap replicates were sequenced separately. In total, we extracted DNA from 152 microbial trap samples (3 transects × 3 locations × 2 deployed soil types × 2 timepoints × 4 replicates and 8 additional samples from the CCC location) and 57 bulk soil samples (3 transects × 3 locations × 2 timepoints × 3 replicates and 3 additional replicates from the CCC location).

### Amplicon sequencing and amplicon cleanup

Microbial composition was characterized with amplicon sequencing of the 16S rRNA gene (515F and 806R) [39, 40] and fungal ITS1 region (ITS1F and 58A2R) [41, 42]. The PCR mixes for both reactions were as follows: Twelve microliters of 5Prime HotMasterMix, 1.5 µL of each primer (10 µM), 1.5 µL template DNA, and 13.5 µL molecular grade water for a final PCR volume of 30 µL. Bacterial 16S rRNA gene PCR cycling conditions were as follows: 3 min at 94 °C, 25 cycles of 45 s at 94 °C, 60 s at 50 °C and 90 s at 72 °C, and a final elongation step of 10 min at 72 °C. Fungal ITS PCR cycling conditions were as follows: 3 min at 94 °C, 35 cycles of 20 s at 94 °C, 30 s at 45 °C and 45 s at 72 °C, and a final elongation step of 5 min at 72 °C. The resulting amplicons were cleaned using Mag-Bind TotalPure NGS magnetic beads (Omega Bio-Te; catalog: M1378-01). Cleaned amplicons were sent to the Pennsylvania State University Genomics Core Facility (Huck Institutes for the Life Sciences) for indexing, normalization, and sequencing on the Illumina MiSeq sequencing platform (2 × 250 bp). Raw data files in FASTQ format were deposited in the NCBI sequence read archive under Bioproject number PRJNA804562.

### Sequence analysis

Raw demultiplexed 16S rRNA gene and fungal ITS data were processed using the Quantitative Insights into Microbial Ecology (QIIME 2 version 2020.11) pipeline [43]. Briefly, paired-ended 16S rRNA and ITS DNA sequences were imported and trimmed, and denoised using DADA2, which also removes chimeric sequences [44]. The classify-sklearn qiime feature classifier was used to assign taxonomy against the Silva v138 [45] or UNITE v04.02.2020 database [46] at the single nucleotide threshold (ZOTUs; zero-radius OTUs). The dataset was further cleaned by removing sequences identified as chloroplasts or mitochondria, and by removing ZOTUs with less than 115 (0.002%) and 42 (0.001%) sequences for the 16S rRNA and ITS gene datasets, respectively. The cleaned 16S rRNA gene and fungal ITS data were then rarefied at 11,011 and 3,510 sequences per sample, respectively (Supplementary Fig. 2).

### Statistical analysis

Processed sequencing data were imported into the R statistical environment [47] and used to create a Phyloseq object [48]. To compare microbial composition between deployed locations and deployed soil types, a non-metric multidimensional scaling analysis (NMDS) and Principal Coordinates Analysis with a Bray–Curtis dissimilarity index was used. Ordinations were performed using the ordinate function in the Phyloseq package. Patterns elucidated by ordination were tested statistically using Adonis (PERMANOVA) from the vegan package with 999 permutations [49]. To explore the similarity of microbial composition in local bulk and recolonized soils, we extracted Bray–Curtis dissimilarity values for local bulk vs. recolonized soil in individual locations and statistically compared them with a Kruskal–Wallis test in the stats package [47] followed by Dunnett’s post hoc test in the FSA package [50]. As we identified a strong within-location influence on microbial compositions (see “Results”), we only selected Bray–Curtis dissimilarity values for individual transect deployments (e.g. Location 1 - Transect 1 recolonized soil vs. Location 1 - Transect 1 bulk soil). P-values were adjusted using the false-discovery rate to account for multiple testing. To examine the impact of transformation on our dataset, we also performed a center-log-ratio (CLR) transformation on the unrarefied data using the microbiome package [51]. CLR transformed data were used with a Principal Components Analysis with a Euclidean distance and data were compared using Adonis with 999 permutations [49].

## Results

### Active microbial pools are distinct between deployment locations

**Fig. 1:**
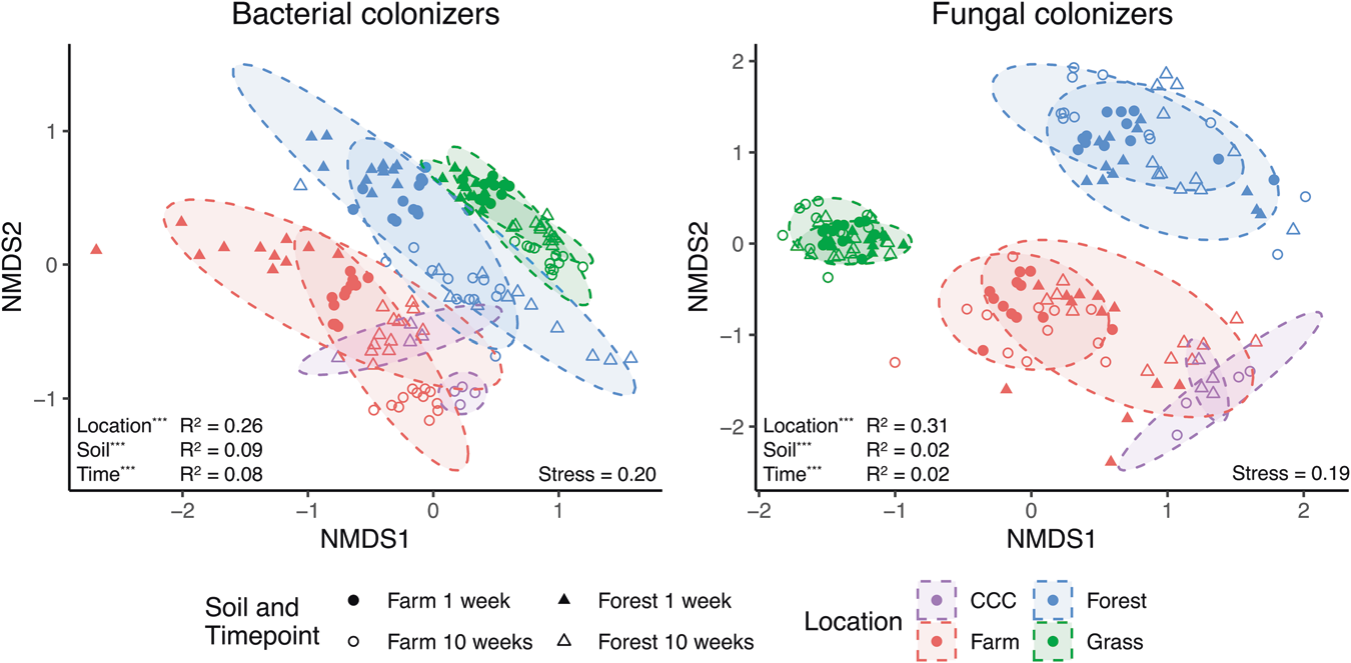
Non-metric multidimensional scaling (NMDS) ordinations of active bacterial (16S rRNA gene) and fungal (ITS) colonizer composition. Samples are colored by location and deployed soil type are different shapes. CCC is the organically managed agricultural field and only includes the 10-week timepoint. 90 % ellipses on the locations are shown. The two individual timepoints are shown as solid or hollow shapes. Ordinations separated by deployed soil type and timepoint are displayed as Supplementary Fig. 2. *** is *p* ≤ 0.001.

Bacterial and fungal colonizer composition was distinct at each deployment location (Fig. 1; Supplementary Fig. 3; Bacteria *F*_3,147_ = 29, *R*^2^ = 0.26, *p* ≤ 0.001; Fungi *F*_3,147_ = 25, *R*^2^ = 0.31, *p* ≤ 0.001; Supplementary Tables 3, 4, 5 and 6), with microbial colonizers at the grass location explaining more of the compositional variance. When comparing the farm and CCC locations with the deployed farm soil type, fungal colonizers were only marginally significant (*F*_1,14_ = 2, *R*^2^ = 0.14, *p* = 0.04) and explained far less compositional variance relative to bacterial colonizers (*F*_1,14_ = 10, *R*^2^ = 0.42, *p* ≤ 0.001; Supplementary Table 6). Overall, deployed soil type was also a significant determinant of bacterial (*F*_1,147_ = 30, *R*^2^ = 0.09, *p* ≤ 0.001) and fungal (*F*_1,147_ = 5, *R*^2^ = 0.02, *p* ≤ 0.001) composition. When comparisons between deployed soil type were performed at each location and timepoint, bacterial, but not fungal, composition was significantly different with every comparison and they typically explained more of the compositional variance (Supplementary Table 7).

### Active microbial pools between transects and deployment sites

In addition to the location influence on active microbial pools, we also observed significant differences in active microbial pools within each location (between transects; Supplementary Figs. 4 and 5). When deployment sites within a transect were grouped together (i.e. transects A, B and C), we saw significant differences for both bacterial and fungal colonizers (16S: *F*_2,140_ = 3, *R*^2^ = 0.04, *p* ≤ 0.001. ITS: *F*_2,140_ = 4, *R*^2^ = 0.05, *p* ≤ 0.001). Comparisons between deployment sites explained 35 % and 43 % of the bacterial and fungal composition variance, respectively, in our dataset (16S: *F*_8,134_ = 12, *R*^2^ = 0.35, *p* ≤ 0.001. ITS: *F*_8,134_ = 14, *R*^2^ = 0.43, *p* ≤ 0.001) and we observed significant differences for just about every location for both timepoints (Supplementary Tables 8 and 9). We also observed significant interactions between deployment site and deployed soil type for bacterial (*F*_8,125_ = 3, *R*^2^ = 0.10, *p* ≤ 0.001) and fungal (*F*_8,125_ = 2, *R*^2^ = 0.07, *p* ≤ 0.001) composition.

**Fig. 2:**
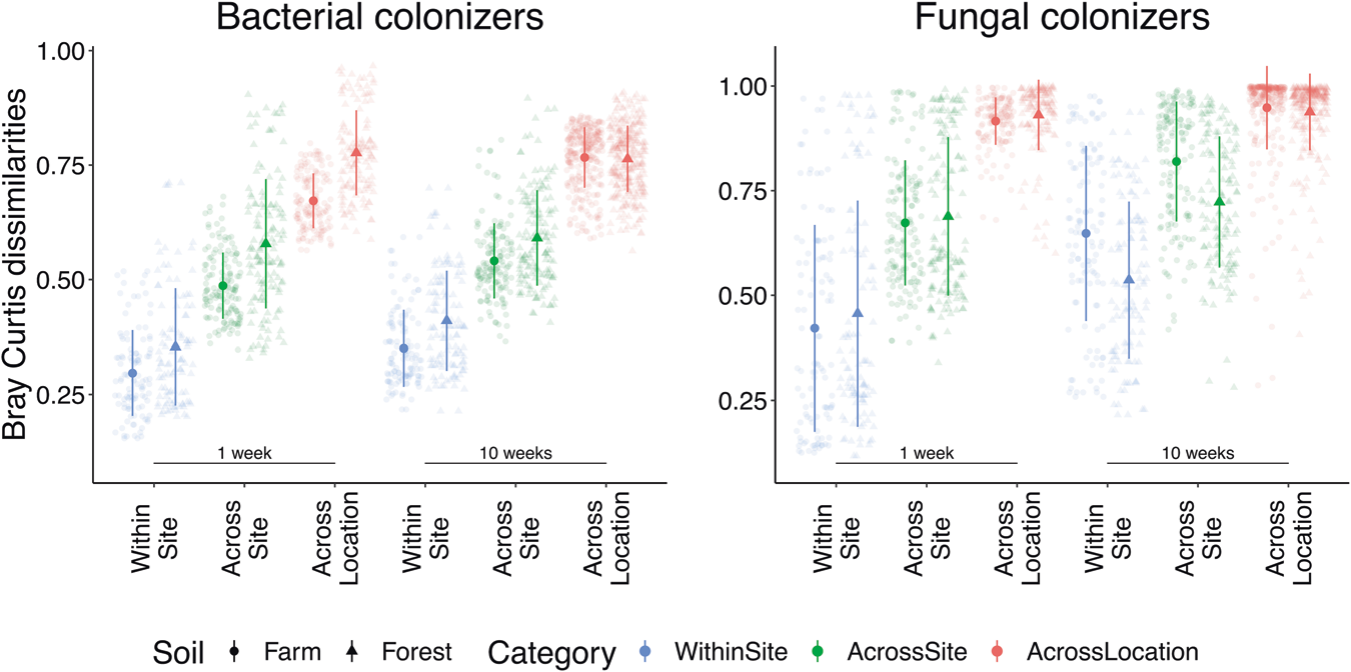
Dot plot to identify how scale impacts active colonizers. Data are Bray–Curtis dissimilarities of replicates within-site, across-sites (between transects within a location) and across locations (between locations within a transect). Lower on the y-axis means greater similarity. Data are mean ± standard deviation. Samples from the CCC organically managed agricultural field were included in within-site and across-location comparisons, but not across-site as only one microbial trap (i.e. one transect) was deployed as a reference plot.

To examine how spatial scale impacts microbial recolonization, we extracted Bray–Curtis dissimilarity values for individual recolonized soils at three scales: within-site (e.g. replicates within a plate), across-sites (between transects within a location; e.g. Transect A Farm vs. Transect B Farm), and across locations within a transect (e.g. Transect A Farm vs. Transect A Forest; Fig. 2). Within-site replicates of fungal colonizers (Bray–Curtis dissimilarity values;1 week: 0.44; 10 weeks: 0.59) were significantly higher than the within-site replicates of bacterial colonizers (1 week: 0.32 and 10 weeks: 0.38) for both timepoints (Kruskal test; 1 week: *H* = 9, d.f. = 1, *p* = 0.002; 10 weeks: H = 129, d.f. = 1, *p* < 0.001). A comparable pattern was observed when contrasting bacterial (1 week: 0.53; 10 weeks: 0.57) and fungal (1 week: 0.68; 10 weeks: 0.77) colonizers across-sites (Kruskal test; 1 week: H = 117, d.f. = 1, *p* < 0.001; 10 weeks: *H* = 219, d.f. = 1, *p* < 0.001). When comparing replicate-dissimilarities across locations, fungal colonizers were almost completely dissimilar from each other (1 week: 0.92; 10 weeks: 0.94; Supplementary Fig. 6), compared to bacterial colonizers (1 week: average = 0.72, *H* = 320, d.f. = 1, *p* < 0.001; 10 weeks: average = 0.77, H = 656, d.f. = 1, *p* < 0.001).

### Comparisons of active colonizers to local bulk soil microorganisms

**Fig. 3:**
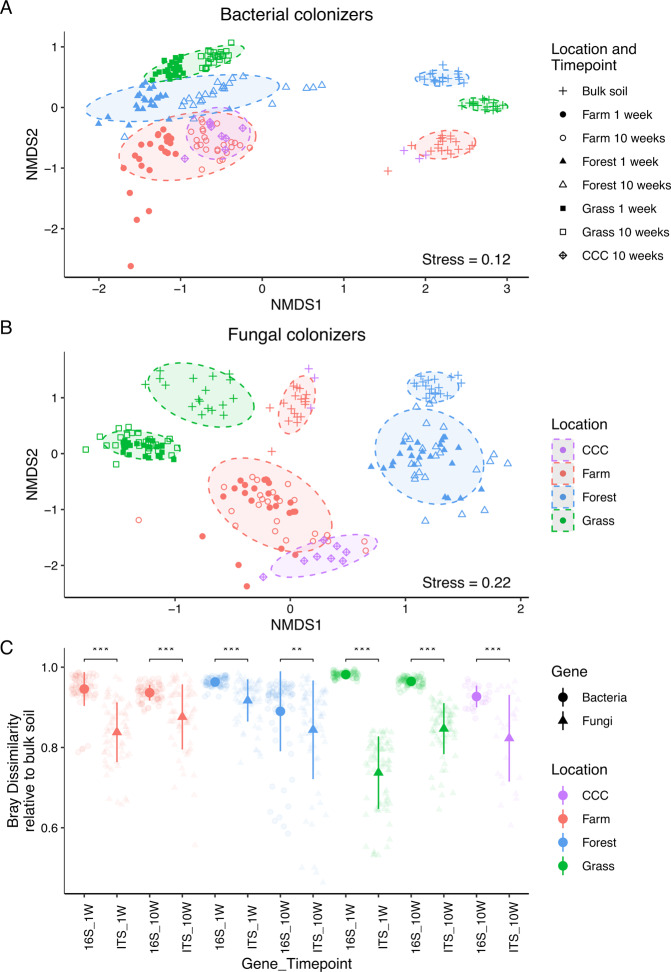
Active microbial colonizer ordinations and Bray-dissimilarities relative to bulk soil. NMDS ordinations of active microbial colonizer and local bulk soil microbial composition (Panels **A**, **B**). Samples are colored by location, with shapes representing different soil sources. The two individual timepoints are shown as solid or hollow shapes. Panel **C** is a dot plot of Bray–Curtis dissimilarity values of local bulk soil relative to recolonized soil at each location and timepoint. Data are mean ± standard deviation. Only comparisons within a transect were chosen because of the transect-level influence on active colonizers. Statistical comparisons were performed per location (Supplementary Table 10) and comparisons between microbial composition at individual timepoints are displayed. Lower on the y-axis means greater similarity between recolonized and local bulk soil. Q values: *** = ≤0.001, ** = ≤0.01, * = ≤0.05.

We hypothesized that active microbial colonizers would reflect local sources (i.e. local bulk soil) of microorganisms. NMDS ordinations indicated closer clustering of fungal composition in bulk and recolonized soils than we observed for bacteria (Fig. 3 and Supplementary Fig. 7). Accordingly, comparisons of local bulk to recolonized soils explained far less compositional variance for fungi (1 week: *F*_1,97_ = 8, *R*^2^ = 0.08, *p* ≤ 0.001. 10 weeks: *F*_1,107_ = 6, *R*^2^ = 0.05, *p* ≤ 0.001) relative to bacteria (1 week: *F*_1,96_ = 39, *R*^2^ = 0.29, *p* ≤ 0.001. 10 weeks: *F*_1,108_ = 32, *R*^2^ = 0.23, *p* ≤ 0.001; Supplementary Table 10). Comparisons of extracted Bray–Curtis dissimilarities between local bulk and recolonized soils identified active fungal colonizers as being more similar to local bulk soil at both 1 week (bacterial mean Bray–Curtis value = 0.96 versus fungal 0.83; *Z* = 18, *q* < 0.001) and 10 weeks (bacterial mean Bray–Curtis value = 0.93 versus fungal 0.85, *Z* = 10, *q* < 0.001; Fig. 3). Accordingly, at every location and for every timepoint, fungal colonizers were more similar to local bulk soil fungi than bacterial colonizers were to local bulk soil bacteria (Supplementary Table 11). In the bulk soils, bacteria had greater Chao1 richness (bacterial average: 416; fungal average: 165) and Shannon’s diversity (bacteria average: 5.6; fungal average: 3.8) when compared to fungi (richness: H = 54, d.f. = 1, *p* < 0.001; diversity: *H* = 54, d.f. = 1, *p* < 0.001).

### Core colonizers

To determine whether we could identify sequences assigned to consistent colonizers (i.e. those found in 100 % of samples), we examined the composition of core colonizers. For the bacterial sequences, three ZOTUs were identified at the 100 % presence threshold across all recolonized soil samples at the 1 week timepoint; the ZOTUs were assigned to the *Pedobacter* and *Allorhizobium-Neorhizobium-Pararhizobium-Rhizobium* genera (ZOTUs 59f5b8 and a15b0f, respectively), and the *Enterobacteriaceae* family (ZOTU 154780). A BLAST search identified the *Allorhizobium-Neorhizobium-Pararhizobium-Rhizobium* genus sequence as an unknown *Rhizobium* species. At the 10-week timepoint, bacterial sequences assigned to the *Devosia* genus (ZOTU cfd199) and the unknown *Rhizobium* species (ZOTU a15b0f) were found at the 100 % threshold. No core colonizers were identified for fungal composition at the 100 % threshold, but at the 90 % threshold sequences assigned to *Hannaella zeae* and *Cladosporium delicatulum* (ZOTUs 07a987 and c01238, respectively) were identified at the 1 week timepoint. We examined core genera (100 % presence) at each location at the 1 week timepoint to identify whether specific genera were consistently recolonizing our deployed soils. The Forest and Grass locations had the greatest overlap of core-colonizing bacterial genera (Supplementary Table 12) and the Grass location had the greatest number of core-colonizing fungal genera (Supplementary Fig. 13). The *Massilia*, *Stenotrophomonas* and *Pseudomonas* genera had the greatest relative abundance of the core bacterial genera in the Farm (9 % average relative abundance), Grass (12 %) and Forest (13 %) locations, respectively. The *Fusarium*, *Pyrenochaetopsis* and *Cladosporium* genera had the greatest relative abundance of the core fungal genera in the Farm (24 %), Grass (43 %) and Forest (24 %) locations, respectively.

## Discussion

There is increasing interest in managing soil microorganisms in agricultural settings, both through active and passive management approaches. However, rational management requires that we understand how existing soil microbial pools are spatially structured, and how environmental constraints shape the composition of active, or potentially active, bacteria and fungi throughout the system. The microorganisms that colonize agricultural soils and plants can originate from many sources, so active microbial pools might be driven by microbial influx that is controlled at a broad scale (e.g. wind; rain), a narrow scale (e.g. differences in adjoining land types), and/or a fine scale (e.g. local differences in soil/plant conditions). Our overall question was whether impactful microbial management is even possible or does broad scale dispersal overwhelm farm-scale management decisions? In this study, we deployed microbial traps, containing two distinct soil types, in transects spanning three adjoining locations (farmland; managed grass strip; contiguous forest) to assess how varying spatial scales shape microbial recolonization and community assembly dynamics.

We identified substantial across-location (i.e. broad; locations with different land use) and within-location (i.e. fine scale; within sites with the same land use) differences in active colonizer composition, suggesting differences in microbial recolonization capacity and community development at fine and broad scales. Consistent with our data, spatial influences on microbial influx have been reported previously [14, 52–55] and the distribution and composition of microorganisms are influenced by biogeography [12, 13]. Our data indicate that the “active microbial seed banks”, those microbes that are available to colonize, differ in neighboring locations at all scales, suggesting that the surrounding environment plays a strong role in driving microbial influx. When comparing microbial influx of bacterial and fungal colonizers, location had a stronger influence on fungal colonizer composition relative to bacterial colonizers (Fig. 2). Previous studies have identified fungi as being more dispersal-limited than bacteria in flowers [56] and plant rhizosphere environments [57], and in agreement, our data indicate that fungal colonizers were almost completely dissimilar across environments (Fig. 2). Relative to fungi, the greater similarity in bacterial colonizer composition within-site, across-transects, and across locations, may indicate the mixing of bacteria between neighboring locations [26]. The stronger influence of location on active fungal composition may indicate that the retention of biodiversity/forest patches (i.e. farmscaping) could be important for maintaining reservoirs of fungal diversity.

While we expected to observe differing patterns of microbial influx across distal locations, we also observed differences in active colonizers within a location (between transects) and over as little as 20 m distance. Previously, Dickie et.al [55] observed differences in ectomycorrhizal dispersal at around 15 m from forest edges and Bell [52] observed bacterial dispersal differences at 71 m in tree-holes. Generally, microbial composition was 0.53–0.77 (Bray–Curtis dissimilarity values) dissimilar to each other among transects within a location. We did observe slight differences in soil physiochemical properties within a location (i.e. between transects; Supplementary Table 1), which may have impacted the bulk soil active microbial pool between transects and may explain the high degree of between-transect heterogeneity between microbial colonizers. Differences in active microbial composition within a location (i.e. a single land use area; between transects) may impact plant-driven microbial recruitment patterns from local microbial pools and could indicate microbial-driven competitive exclusion of microorganisms. Compositional differences between replicates within a site were responsible for roughly 30–40 % of the dissimilarity, which could be due to a combination of priority effects driving slightly differing assembly trajectories [58, 59] and stochasticity.

We deployed two soil types to investigate the role of edaphic filtering of microbial colonizers. In our data, deployed soil type significantly impacted microbial colonizer composition, but explained more of the compositional variance of bacterial colonizers relative to fungal colonizers (Supplementary Table 7). These differences were most apparent at the 1 week timepoint, where deployed soil type significantly impacted bacterial colonizers at all locations but only impacted fungal colonizers at the farm location. While differences between deployed soil types were observed at 10 weeks for fungal colonizers, this could indicate that early fungal colonizers are more tolerant of different abiotic pressures before individual communities set on different developmental trajectories. Previous studies suggest that bacterial and fungal assembly are predominantly structured by different abiotic constraints. Bacteria are more strongly structured by soil pH [12, 60], while fungi are more influenced by factors like precipitation [12], with fungi apparently less constrained by environmental factors relative to bacteria [8]. In agreement, our data indicates that bacterial colonizer composition was more strongly shaped by edaphic filtering than fungal colonizer composition which could have been due to differences in the pH of our two deployed soil types. Soil pH is one of the principal drivers of bacterial compositions [12, 60], which was a considering factor when selecting our initial soils. As fungal colonizers appeared to be more tolerant of abiotic constraints in the deployed soils, agricultural soil management practices that alter soil properties and habitat space, such as tillage [24], may have a greater influence on the active bacterial colonizers at a given site relative to active fungi. For example, in our dataset, frequently tilled soils (CCC) and soils with years of conventional management using conservation tillage (Farm) had more similar fungal colonizer compositions relative to bacterial colonizer compositions between both soils.

Passive management approaches in agriculture have employed intercropping and farmscaping to shape farm-scale biodiversity. Conventional agricultural management practices can deplete microbial function and diversity [19–23] and transitions to organic management practices are thought to promote soil biodiversity and reduce farmer needs for synthetic inputs [61]. Previous studies have identified improved microbial abundance, function, and differences in microbial composition, in organically managed systems relative to conventionally managed systems [61–63]. In our data, we also identified differences in both bacterial and fungal colonizer composition when comparing our Farm and CCC locations. However, this difference was greater for bacterial colonizers, with only marginal significance between Farm and CCC locations identified for fungal colonizers. Notably, a previous study identified a 20-year legacy effect of conventional management on arbuscular mycorrhizal fungi abundance [64]. While bacteria were not included, this study may indicate that fungi are sensitive to legacy effects and could explain the more similar fungal colonizer composition between our Farm (low tillage) and CCC (high tillage) locations.

We hypothesized that microbial colonizer composition would reflect the local sources of microorganisms found bulk soil immediately adjacent to each microbial trap. In contrast, both fungal and bacterial colonizer composition were significantly different from the composition of local bulk soil microorganisms; though, fungal colonizers shared a greater degree of compositional overlap with local bulk soil fungi relative to bacterial colonizers and local bulk soil bacteria. The large degree of dormancy in soil microbiomes [1–4] and the often large quantity of legacy DNA [5] can mask which microorganisms are active in bulk soil. While differences between active and bulk soil microorganisms have been shown before [1, 4], in our data, we have shown that fungal colonizers are a better representation of the local bulk soil fungi than bacterial colonizers are of local bulk soil bacteria. The differences between active bacterial and fungal representation in bulk soil may be due to various factors, including: (i) differences in the soil microbial seed bank, (ii) fungal mycelial networks, and (iii) differences in short range dispersal capacity. As bacteria have greater abundance and diversity in soil than fungi, and as shown by a greater richness and diversity of bacteria in our bulk soils relative to fungi, an active colonizing proportion may represent a smaller fraction of the overall bacterial seed bank driving a large difference between bulk and active bacterial composition. In addition, since fungi are able to grow in networks, fungi from outside the microbial traps may be able to extend hyphae within the microbial trap allowing them to more easily explore and tolerate differing abiotic conditions.

In this study, we developed a means of reliably deploying sterile soils with sustained soil-to-soil contact to examine recolonization dynamics at differing spatial scales. Few studies have used an approach like this, so many questions remain on how changes to this design could impact observations. One key consideration for future studies is that season is likely to have substantial impacts on community development, as has been shown a number of times in bulk soils (e.g. [65]). Although season is likely to change the specific dispersing pool, we hypothesize that the community-level location differences observed in our data would be apparent across seasons, although perhaps the extent of plant growth would influence the number and type of dispersing microorganisms. Another consideration is sampling date following deployment. We chose 1 week and 10 weeks to capture both pioneer and secondary colonizers, but additional timepoints would inform on the stability of community development over time. For our microbial traps, we expected the 18 µm nylon membrane to allow the vast majority of microorganisms to traverse the membrane and colonize the deployed soils and this was also intended to mirror the membrane size used by Albright and Martiny [11]. Future studies may consider including membranes with greater and smaller pore sizes to fractionate colonizers and to allow larger organisms, such as protists, to enter and apply predation pressure. In a more simplified microbial trap construction, the use of nylon bags or stockings could be an adequate replacement.

We contrasted colonization patterns for an active proportion of the bacterial and fungal soil seed bank to understand how spatial scales and abiotic constraints shape community assembly dynamics and microbial recolonization capacity. Our data indicated that location (i.e. farmland; managed grass strip; contiguous forest) shaped both fungal and bacterial colonizer composition but had a stronger influence on fungal colonizers. Likewise, both fungal and bacterial colonizers were shaped by the soil type contained within the microbial traps, but deployed soil type had a greater influence on bacterial colonizers. Comparisons between our conventionally- and organically managed farms identified bacterial colonizers as being more strongly shaped by these management practices than fungal colonizers. Effective microbial management in agricultural settings requires an understanding of what microorganisms are active and available and how environmental factors and management practices can shape active microbial pools. Here, we have created a reproducible method for capturing active microbial colonizers to contrast patterns in active bacterial and fungal assembly. These data are important for determining the relative importance of direct soil management, landscape management, and targeted microbial introductions in shaping agricultural microbiomes.

## Supplementary information

Supplementary information
