# Supplementary material for: Farm-scale differentiation of active microbial colonizers
Source: ISME Commun. 2022 Apr 20;2:39. doi: 10.1038/s43705-022-00120-9 (PMC9723676; doi:10.1038/s43705-022-00120-9)
Supplement: Supplementary file 1 — Supplementary information [file 43705_2022_120_MOESM1_ESM.pdf]

Farm-scale differentiation of active microbial colonizers

William L King[1], Laura M Kaminsky[1], Sarah C Richards[1,2,3], Brosi A Bradley[4], Jason P Kaye[2,4], Terrence H Bell[1,2,3*]

1 - Department of Plant Pathology and Environmental Microbiology, The Pennsylvania State University, University Park, PA, 16802, USA

2 - Intercollege Graduate Degree Program in Ecology, The Pennsylvania State University, University Park, PA, 16802, USA

3 - Intercollege Graduate Degree Program in International Agriculture and Development, The Pennsylvania State University, University Park, PA, 16802, USA

4 - Department of Ecosystem Science and Management, The Pennsylvania State University, University Park, PA, 16802, USA

*Correspondence:

Postal address: The Pennsylvania State University, 317 Buckhout Lab, University Park, PA, USA, 16802

Telephone number: 814-865-9653

Email: thb15@psu.edu

Supplementary Table 1: Soil analysis. Provided data includes pH, total nitrogen (N), total carbon (C), phosphorous (P), potassium (K), magnesium (Mg), calcium (Ca), organic matter (OM), zinc (Zn), copper (Cu) and sulfur (S). All units are parts per million (ppm) unless specified. Soil for deployment was collected from a farm and forest. Soil was deployed into a farm, grassy and forest location, and into an organically managed farm location (CCC). See Supplementary Table 2 for transect (A, B and C) locations.

Soil	pH	N (%)	C (%)	P	K	Mg	Ca	OM (%)	Zn	Cu	S	Textural class
Farm*	6.3	0.14	1.34	17	126	115	1037	2.2	1.6	5.7	9.8	Clay loam
Forest*	5.2	0.59	7.34	12	158	220	735	12.6	6.2	1.5	12.7	Loam
Sterile Farm	6.7	0.09	0.92	34	114	67	923	1.8	1.0	2.5	24.8	Loam
Sterile Forest	5.6	0.72	7.01	52	131	154	735	8.4	2.6	1.0	56.4	Sandy loam
A1_Farm	6.6	0.22	2.05	48	108	159	1063	3.4	1.4	0.9	6.7	Silty clay loam
A2_Grass	5.8	0.40	3.64	32	112	107	583	6.0	2.5	1.0	9.6	Silt loam
A3_Forest	5.8	0.52	7.30	102	120	94	1435	10.3	4.3	1.2	13.5	Silt loam
B1_Farm	7.0	0.19	1.88	56	104	153	980	3.2	2.3	1.0	9.8	Silty clay loam
B2_Grass	6.0	0.31	2.78	14	79	77	569	4.7	1.9	1.1	7.9	Silt loam
B3_Forest	6.1	0.45	6.16	53	104	97	1083	10.0	3.5	0.9	9.6	Loam
C1_Farm	6.9	0.18	1.81	32	98	140	740	3.0	1.0	0.8	6.1	Clay loam
C2_Grass	5.8	0.30	2.66	22	78	92	492	4.5	1.6	0.9	8.2	Loam
C3_Forest	5.6	0.57	7.47	39	130	102	947	11.3	3.9	1.0	9.3	Loam
CCC	6.8	0.15	1.47	32	135	137	934	2.5	1.8	5	7.5	Clay loam

*Soil was analyzed before sand addition

Supplementary Table 2: Transect deployment locations. All sites within and between transects were 20 m apart. All transects were deployed at the Pennsylvania State University at the Russell E. Larson Agricultural Research Center (40°42'53.0"N 77°55'51.2"W). CCC plots are the organically managed portion of the Research Center.

Deployment	Transect A	Transect B	Transect C
Agricultural farm	40°42'53.2"N 77°55'51.5"W	40°42'53.5"N 77°55'50.8"W	40°42'53.7"N 77°55'50.1"W
Grassy intermediary	40°42'52.6"N 77°55'51.1"W	40°42'52.8"N 77°55'50.4"W	40°42'53.0"N 77°55'49.7"W
Contiguous forest	40°42'52.0"N 77°55'50.9"W	40°42'52.2"N 77°55'50.1"W	40°42'52.4"N 77°55'49.2"W
CCC plots	40°43'17.6"N 77°55'40.2"W		

Supplementary Table 3: PERMANOVA comparisons between transformation methods. Shown data include rarefied data compared with a Bray-Curtis dissimilarity index and unrarefied center-log-transformed (clr) data with Euclidean distances.

Gene	Factor	Bray-Curtis rarefied data	Euclidean clr data
16S	Location	df = 3, F = 29, R2 = 0.26, $p \leq 0.001$	df = 3, F = 19, R2 = 0.22, $p \leq 0.001$
	Soil	df = 1, F = 30, R2 = 0.09, $p \leq 0.001$	df = 1, F = 16, R2 = 0.06, $p \leq 0.001$
	Timepoint	df = 1, F = 25, R2 = 0.08, $p \leq 0.001$	df = 1, F = 15, R2 = 0.06, $p \leq 0.001$
	Location:Soil	df = 3, F = 7, R2 = 0.06, $p \leq 0.001$	df = 3, F = 5, R2 = 0.05, $p \leq 0.001$
	Location:Timepoint	df = 2, F = 8, R2 = 0.05, $p \leq 0.001$	df = 2, F = 6, R2 = 0.05, $p \leq 0.001$
	Soil:Timepoint	df = 1, F = 7, R2 = 0.02, $p \leq 0.001$	df = 1, F = 4, R2 = 0.02, $p \leq 0.001$
	Location:Soil:Timepoint	df = 2, F = 4, R2 = 0.02, $p \leq 0.001$	df = 2, F = 3, R2 = 0.02, $p \leq 0.001$
	Residuals	df = 137	df = 137
	Total	df = 150	df = 150
ITS	Location	df = 3, F = 25, R2 = 0.31, $p \leq 0.001$	df = 3, F = 23, R2 = 0.29, $p \leq 0.001$
	Soil	df = 1, F = 5, R2 = 0.02, $p \leq 0.001$	df = 1, F = 5, R2 = 0.02, $p \leq 0.001$
	Timepoint	df = 1, F = 6, R2 = 0.02, $p \leq 0.001$	df = 1, F = 7, R2 = 0.03, $p \leq 0.001$
	Location:Soil	df = 3, F = 3, R2 = 0.03, $p \leq 0.001$	df = 3, F = 3, R2 = 0.04, $p \leq 0.001$
	Location:Timepoint	df = 2, F = 4, R2 = 0.04, $p \leq 0.001$	df = 2, F = 5, R2 = 0.04, $p \leq 0.001$
	Soil:Timepoint	df = 1, F = 2, R2 = 0.007, p = 0.06	df = 1, F = 2, R2 = 0.008, p = 0.01
	Location:Soil:Timepoint	df = 2, F = 1, R2 = 0.01, p = 0.07	df = 2, F = 2, R2 = 0.01, p = 0.02
	Residuals	df = 137	df = 137
	Total	df = 150	df = 150

Supplementary Table 4: PERMANOVA location comparison with grouped soil-types

Timepoint	Gene	Overall	Location (grouped soil-type)
1 week	16S rRNA	$F_{2,68} = 17$ $R^2 = 0.33$ $p \leq 0.001$	Farm vs. Grass: $F_{1,45} = 19$, $R^2 = 0.30$, $p \leq 0.001$
			Farm vs. Forest: $F_{1,45} = 15$, $R^2 = 0.25$, $p \leq 0.001$
			Forest vs. Grass: $F_{1,46} = 16$, $R^2 = 0.26$, $p \leq 0.001$
	ITS	$F_{2,69} = 23$ $R^2 = 0.40$ $p \leq 0.001$	Farm vs. Grass: $F_{1,46} = 25$, $R^2 = 0.35$, $p \leq 0.001$
			Farm vs. Forest: $F_{1,46} = 13$ $R^2 = 0.22$, $p \leq 0.001$
			Forest vs. Grass: $F_{1,46} = 35$, $R^2 = 0.43$, $p \leq 0.001$
10 weeks	16S rRNA	$F_{3,76} = 13$ $R^2 = 0.33$ $p \leq 0.001$	Farm vs. Grass: $F_{1,46} = 19$, $R^2 = 0.30$, $p \leq 0.001$
			Farm vs. Forest: $F_{1,46} = 11$, $R^2 = 0.20$, $p \leq 0.001$
			Forest vs. Grass: $F_{1,46} = 12$, $R^2 = 0.21$, $p \leq 0.001$
			Farm vs. CCC: $F_{1,30} = 8$, $R^2 = 0.22$, $p \leq 0.001$
			Grass vs. CCC: $F_{1,30} = 14$, $R^2 = 0.32$, $p \leq 0.001$
			Forest vs. CCC: $F_{1,30} = 8$, $R^2 = 0.22$, $p \leq 0.001$
	ITS	$F_{3,75} = 11$ $R^2 = 0.31$ $p \leq 0.001$	Farm vs. Grass: $F_{1,45} = 18$, $R^2 = 0.29$, $p \leq 0.001$
			Farm vs. Forest: $F_{1,46} = 7$, $R^2 = 0.13$, $p \leq 0.001$
			Forest vs. Grass: $F_{1,46} = 17$, $R^2 = 0.26$, $p \leq 0.001$
			Farm vs. CCC: $F_{1,29} = 4$, $R^2 = 0.13$, $p \leq 0.001$
			Grass vs. CCC: $F_{1,30} = 16$, $R^2 = 0.35$, $p \leq 0.001$
			Forest vs. CCC: $F_{1,30} = 6$, $R^2 = 0.18$, $p \leq 0.001$

Supplementary Table 5: PERMANOVA location comparison with individual soil-types for week 1

Timepoint	Gene	Soil-type	Overall	Location (individual soil-type)
1 week	16S rRNA	Farm	$F_{2,33} = 16$<br>$R^2 = 0.50$<br>$p \leq 0.001$	Farm vs. Grass: $F_{1,22} = 20$, $R^2 = 0.48$, $p \leq 0.001$<br>Farm vs. Forest: $F_{1,22} = 15$, $R^2 = 0.41$, $p \leq 0.001$<br>Forest vs. Grass: $F_{1,22} = 14$, $R^2 = 0.39$, $p \leq 0.001$
		Forest	$F_{2,32} = 13$<br>$R^2 = 0.45$<br>$p \leq 0.001$	Farm vs. Grass: $F_{1,21} = 16$, $R^2 = 0.43$, $p \leq 0.001$<br>Farm vs. Forest: $F_{1,21} = 10$, $R^2 = 0.32$, $p \leq 0.001$<br>Forest vs. Grass: $F_{1,22} = 14$, $R^2 = 0.39$, $p \leq 0.001$
	ITS	Farm	$F_{2,33} = 14$<br>$R^2 = 0.45$<br>$p \leq 0.001$	Farm vs. Grass: $F_{1,22} = 16$, $R^2 = 0.41$, $p \leq 0.001$<br>Farm vs. Forest: $F_{1,22} = 9$, $R^2 = 0.29$, $p \leq 0.001$<br>Forest vs. Grass: $F_{1,22} = 18$, $R^2 = 0.44$, $p \leq 0.001$
		Forest	$F_{2,33} = 12$<br>$R^2 = 0.43$<br>$p \leq 0.001$	Farm vs. Grass: $F_{1,22} = 13$, $R^2 = 0.38$, $p \leq 0.001$<br>Farm vs. Forest: $F_{1,22} = 7$, $R^2 = 0.23$, $p \leq 0.001$<br>Forest vs. Grass: $F_{1,22} = 21$, $R^2 = 0.49$, $p \leq 0.001$

Supplementary Table 6: PERMANOVA location comparison with individual soil-types for week 10

Timepoint	Gene	Soil-type	Overall	Location (individual soil-type)
10 weeks	16S rRNA	Farm	$F_{3,36} = 13$<br>$R^2 = 0.52$<br>$p \leq 0.001$	Farm vs. Grass: $F_{1,22} = 19$, $R^2 = 0.46$, $p \leq 0.001$<br>Farm vs. Forest: $F_{1,22} = 11$, $R^2 = 0.34$, $p \leq 0.001$<br>Forest vs. Grass: $F_{1,22} = 14$, $R^2 = 0.39$, $p \leq 0.001$<br>Farm vs. CCC: $F_{1,14} = 10$, $R^2 = 0.42$, $p \leq 0.001$<br>Grass vs. CCC: $F_{1,14} = 14$, $R^2 = 0.50$, $p \leq 0.001$<br>Forest vs. CCC: $F_{1,14} = 10$, $R^2 = 0.42$, $p \leq 0.001$
		Forest	$F_{3,36} = 10$<br>$R^2 = 0.46$<br>$p \leq 0.001$	Farm vs. Grass: $F_{1,22} = 17$, $R^2 = 0.43$, $p \leq 0.001$<br>Farm vs. Forest: $F_{1,22} = 9$, $R^2 = 0.30$, $p \leq 0.001$<br>Forest vs. Grass: $F_{1,22} = 9$, $R^2 = 0.30$, $p \leq 0.001$<br>Farm vs. CCC: $F_{1,14} = 6$, $R^2 = 0.29$, $p \leq 0.001$<br>Grass vs. CCC: $F_{1,14} = 14$, $R^2 = 0.50$, $p \leq 0.001$<br>Forest vs. CCC: $F_{1,14} = 6$, $R^2 = 0.31$, $p \leq 0.001$
	ITS	Farm	$F_{3,36} = 5$<br>$R^2 = 0.31$<br>$p \leq 0.001$	Farm vs. Grass: $F_{1,22} = 9$, $R^2 = 0.30$, $p \leq 0.001$<br>Farm vs. Forest: $F_{1,22} = 4$, $R^2 = 0.15$, $p \leq 0.001$<br>Forest vs. Grass: $F_{1,22} = 8$, $R^2 = 0.26$, $p \leq 0.001$<br>Farm vs. CCC: $F_{1,14} = 2$, $R^2 = 0.14$, $p = 0.04$<br>Grass vs. CCC: $F_{1,14} = 7$, $R^2 = 0.34$, $p \leq 0.001$<br>Forest vs. CCC: $F_{1,22} = 3$, $R^2 = 0.16$, $p \leq 0.001$
		Forest	$F_{3,35} = 10$<br>$R^2 = 0.45$<br>$p \leq 0.001$	Farm vs. Grass: $F_{1,21} = 13$, $R^2 = 0.38$, $p \leq 0.001$<br>Farm vs. Forest: $F_{1,21} = 5$, $R^2 = 0.20$, $p \leq 0.001$<br>Forest vs. Grass: $F_{1,22} = 15$, $R^2 = 0.40$, $p \leq 0.001$<br>Farm vs. CCC: $F_{1,13} = 4$, $R^2 = 0.25$, $p = 0.002$<br>Grass vs. CCC: $F_{1,14} = 16$, $R^2 = 0.54$, $p \leq 0.001$<br>Forest vs. CCC: $F_{1,14} = 7$, $R^2 = 0.35$, $p = 0.002$

Supplementary Table 7: PERMANOVA comparisons of deployed soil-type comparisons within locations

Timepoint	Gene	Soil-type comparison
1 week	16S rRNA	Farm: $F_{1,21} = 8$, $R^2 = 0.27$, $p \leq 0.001$<br>Forest: $F_{1,22} = 7$, $R^2 = 0.25$, $p \leq 0.001$<br>Grass: $F_{1,22} = 14$, $R^2 = 0.39$, $p \leq 0.001$
	ITS	Farm: $F_{1,22} = 3$, $R^2 = 0.11$, $p = 0.002$<br>Forest: Not significant<br>Grass: Not significant
10 weeks	16S rRNA	Farm: $F_{1,22} = 10$, $R^2 = 0.31$, $p \leq 0.001$<br>Forest: $F_{1,22} = 9$, $R^2 = 0.29$, $p \leq 0.001$<br>Grass: $F_{1,22} = 13$, $R^2 = 0.37$, $p \leq 0.001$<br>CCC: $F_{1,6} = 6$, $R^2 = 0.48$, $p = 0.04$
	ITS	Farm: $F_{1,22} = 3$, $R^2 = 0.12$, $p = 0.02$<br>Forest: $F_{1,22} = 3$, $R^2 = 0.12$, $p \leq 0.001$<br>Grass: $F_{1,22} = 3$, $R^2 = 0.14$, $p = 0.002$<br>CCC: Not significant

Supplementary Table 8: PERMANOVA transect comparisons within locations at the 1-week timepoint.

Gene	Location	Soil-type	Overall	Location (individual soil-type)
16S rRNA	Farm	Farm	$F_{2,9} = 7$<br>$R^2 = 0.59$<br>$p \leq 0.001$	A vs. B: $F_{1,6} = 9$, $R^2 = 0.59$, p = 0.03<br>A vs. C: $F_{1,6} = 6$, $R^2 = 0.50$, p = 0.03<br>B vs. C: $F_{1,6} = 5$, $R^2 = 0.47$, p = 0.03
		Forest	$F_{2,8} = 7$<br>$R^2 = 0.64$<br>$p \leq 0.001$	A vs. B: $F_{1,6} = 6$, $R^2 = 0.50$, p = 0.03<br>A vs. C: $F_{1,5} = 7$, $R^2 = 0.59$, p = 0.02<br>B vs. C: $F_{1,6} = 8$, $R^2 = 0.62$, p = 0.03
	Forest	Farm	$F_{2,9} = 8$<br>$R^2 = 0.65$<br>$p \leq 0.001$	A vs. B: $F_{1,6} = 7$, $R^2 = 0.54$, p = 0.03<br>A vs. C: $F_{1,6} = 7$, $R^2 = 0.53$, p = 0.02<br>B vs. C: $F_{1,6} = 15$, $R^2 = 0.71$, p = 0.03
		Forest	$F_{2,9} = 8$<br>$R^2 = 0.65$<br>$p \leq 0.001$	A vs. B: $F_{1,6} = 6$, $R^2 = 0.50$, p = 0.04<br>A vs. C: $F_{1,6} = 8$, $R^2 = 0.58$, p = 0.03<br>B vs. C: $F_{1,6} = 14$, $R^2 = 0.70$, p = 0.03
	Grass	Farm	$F_{2,9} = 6$<br>$R^2 = 0.58$<br>$p \leq 0.001$	A vs. B: $F_{1,6} = 5$, $R^2 = 0.45$, p = 0.04<br>A vs. C: $F_{1,6} = 8$, $R^2 = 0.57$, p = 0.03<br>B vs. C: $F_{1,6} = 6$, $R^2 = 0.49$, p = 0.02
		Forest	$F_{2,9} = 5$<br>$R^2 = 0.53$<br>p = 0.002	A vs. B: $F_{1,6} = 5$, $R^2 = 0.45$, p = 0.03<br>A vs. C: $F_{1,6} = 5$, $R^2 = 0.47$, p = 0.03<br>B vs. C: $F_{1,6} = 5$, $R^2 = 0.46$, p = 0.03
ITS	Farm	Farm	$F_{2,9} = 2$<br>$R^2 = 0.29$<br>p = 0.03	A vs. B: $F_{1,6} = 2$, $R^2 = 0.28$, p = 0.02<br>A vs. C: $F_{1,6} = 2$, $R^2 = 0.28$, p = 0.03<br>B vs. C: NS
		Forest	$F_{2,9} = 2$<br>$R^2 = 0.28$<br>p = 0.02	A vs. B: $F_{1,6} = 2$, $R^2 = 0.29$, p = 0.04<br>A vs. C: NS<br>B vs. C: $F_{1,6} = 2$, $R^2 = 0.22$, p = 0.03
	Forest	Farm	$F_{2,9} = 7$<br>$R^2 = 0.60$<br>$p \leq 0.001$	A vs. B: $F_{1,6} = 5$, $R^2 = 0.45$, p = 0.04<br>A vs. C: $F_{1,6} = 5$, $R^2 = 0.44$, p = 0.03<br>B vs. C: $F_{1,6} = 24$, $R^2 = 0.80$, p = 0.02
		Forest	$F_{2,9} = 8$<br>$R^2 = 0.64$<br>$p \leq 0.001$	A vs. B: $F_{1,6} = 7$, $R^2 = 0.55$, p = 0.02<br>A vs. C: NS (p = 0.056)<br>B vs. C: $F_{1,6} = 15$, $R^2 = 0.72$, p = 0.03
	Grass	Farm	$F_{2,9} = 18$<br>$R^2 = 0.80$<br>$p \leq 0.001$	A vs. B: $F_{1,6} = 23$, $R^2 = 0.79$, p = 0.03<br>A vs. C: $F_{1,6} = 19$, $R^2 = 0.76$, p = 0.03<br>B vs. C: $F_{1,6} = 12$, $R^2 = 0.66$, p = 0.04
		Forest	$F_{2,9} = 12$<br>$R^2 = 0.74$<br>$p \leq 0.001$	A vs. B: $F_{1,6} = 9$, $R^2 = 0.59$, p = 0.04<br>A vs. C: $F_{1,6} = 14$, $R^2 = 0.71$, p = 0.02<br>B vs. C: $F_{1,6} = 17$, $R^2 = 0.73$, p = 0.03

Supplementary Table 9: PERMANOVA transect comparisons within locations at the 10-week timepoint.

Gene	Location	Soil-type	Overall	Location (individual soil-type)
16S rRNA	Farm	Farm	$F_{2,9} = 8$<br>$R^2 = 0.64$<br>$p \leq 0.001$	A vs. B: $F_{1,6} = 4$, $R^2 = 0.42$, p = 0.03<br>A vs. C: $F_{1,6} = 10$, $R^2 = 0.64$, p = 0.03<br>B vs. C: $F_{1,6} = 10$, $R^2 = 0.63$, p = 0.03
		Forest	$F_{2,9} = 4$<br>$R^2 = 0.48$<br>$p \leq 0.001$	A vs. B: $F_{1,6} = 2$, $R^2 = 0.27$, p = 0.03<br>A vs. C: $F_{1,6} = 6$, $R^2 = 0.49$, p = 0.02<br>B vs. C: $F_{1,6} = 5$, $R^2 = 0.43$, p = 0.03
	Forest	Farm	$F_{2,9} = 6$<br>$R^2 = 0.58$<br>$p \leq 0.001$	A vs. B: $F_{1,6} = 7$, $R^2 = 0.53$, p = 0.04<br>A vs. C: $F_{1,6} = 5$, $R^2 = 0.47$, p = 0.03<br>B vs. C: $F_{1,6} = 6$, $R^2 = 0.50$, p = 0.03
		Forest	$F_{2,9} = 6$<br>$R^2 = 0.58$<br>$p \leq 0.001$	A vs. B: $F_{1,6} = 9$, $R^2 = 0.59$, p = 0.02<br>A vs. C: $F_{1,6} = 4$, $R^2 = 0.39$, p = 0.03<br>B vs. C: $F_{1,6} = 7$, $R^2 = 0.55$, p = 0.03
	Grass	Farm	$F_{2,9} = 7$<br>$R^2 = 0.61$<br>$p \leq 0.001$	A vs. B: $F_{1,6} = 8$, $R^2 = 0.56$, p = 0.03<br>A vs. C: $F_{1,6} = 8$, $R^2 = 0.57$, p = 0.03<br>B vs. C: $F_{1,6} = 5$, $R^2 = 0.47$, p = 0.04
		Forest	$F_{2,9} = 5$<br>$R^2 = 0.51$<br>$p \leq 0.001$	A vs. B: $F_{1,6} = 6$, $R^2 = 0.52$, p = 0.03<br>A vs. C: $F_{1,6} = 4$, $R^2 = 0.37$, p = 0.02<br>B vs. C: $F_{1,6} = 5$, $R^2 = 0.44$, p = 0.03
ITS	Farm	Farm	$F_{2,9} = 3$<br>$R^2 = 0.42$<br>p = 0.003	A vs. B: NS<br>A vs. C: $F_{1,6} = 4$, $R^2 = 0.41$, p = 0.04<br>B vs. C: $F_{1,6} = 5$, $R^2 = 0.44$, p = 0.03
		Forest	$F_{2,8} = 2$<br>$R^2 = 0.36$<br>p = 0.03	A vs. B: NS<br>A vs. C: NS<br>B vs. C: NS
	Forest	Farm	$F_{2,9} = 2$<br>$R^2 = 0.34$<br>$p \leq 0.001$	A vs. B: $F_{1,6} = 2$, $R^2 = 0.24$, p = 0.03<br>A vs. C: $F_{1,6} = 2$, $R^2 = 0.28$, p = 0.03<br>B vs. C: $F_{1,6} = 3$, $R^2 = 0.31$, p = 0.03
		Forest	$F_{2,9} = 4$<br>$R^2 = 0.45$<br>$p \leq 0.001$	A vs. B: $F_{1,6} = 5$, $R^2 = 0.45$, p = 0.03<br>A vs. C: NS<br>B vs. C: $F_{1,6} = 5$, $R^2 = 0.44$, p = 0.03
	Grass	Farm	$F_{2,9} = 6$<br>$R^2 = 0.59$<br>$p \leq 0.001$	A vs. B: $F_{1,6} = 7$, $R^2 = 0.54$, p = 0.03<br>A vs. C: $F_{1,6} = 9$, $R^2 = 0.60$, p = 0.03<br>B vs. C: $F_{1,6} = 4$, $R^2 = 0.43$, p = 0.02
		Forest	$F_{2,9} = 5$<br>$R^2 = 0.53$<br>$p \leq 0.001$	A vs. B: $F_{1,6} = 7$, $R^2 = 0.53$, p = 0.03<br>A vs. C: $F_{1,6} = 6$, $R^2 = 0.50$, p = 0.03<br>B vs. C: $F_{1,6} = 3$, $R^2 = 0.36$, p = 0.03

Supplementary Table 10: PERMANOVA recolonized soil comparisons to bulk soil.

Location	Overall	Comparison to Bulk soil (grouped soil-type)
16S rRNA	$F_{1,96} = 39$<br>$R^2 = 0.29$<br>$p \leq 0.001$	Farm: $F_{1,30} = 20$, $R^2 = 0.40$, $p \leq 0.001$<br>Forest: $F_{1,31} = 29$, $R^2 = 0.48$, $p \leq 0.001$<br>Grass: $F_{1,31} = 44$, $R^2 = 0.59$, $p \leq 0.001$
ITS	$F_{1,97} = 8$<br>$R^2 = 0.08$<br>$p \leq 0.001$	Farm: $F_{1,31} = 6$, $R^2 = 0.16$, $p \leq 0.001$<br>Forest: $F_{1,31} = 10$, $R^2 = 0.24$, $p \leq 0.001$<br>Grass: $F_{1,31} = 13$, $R^2 = 0.30$, $p \leq 0.001$
16S rRNA	$F_{1,108} = 32$<br>$R^2 = 0.23$<br>$p \leq 0.001$	Farm: $F_{1,31} = 21$, $R^2 = 0.40$, $p \leq 0.001$<br>Forest: $F_{1,31} = 18$, $R^2 = 0.37$, $p \leq 0.001$<br>Grass: $F_{1,31} = 34$, $R^2 = 0.52$, $p \leq 0.001$<br>CCC: $F_{1,9} = 10$, $R^2 = 0.53$, $p = 0.008$
ITS	$F_{1,107} = 6$<br>$R^2 = 0.05$<br>$p \leq 0.001$	Farm: $F_{1,30} = 6$, $R^2 = 0.16$, $p \leq 0.001$<br>Forest: $F_{1,31} = 4$, $R^2 = 0.11$, $p \leq 0.001$<br>Grass: $F_{1,31} = 10$, $R^2 = 0.25$, $p \leq 0.001$<br>CCC: $F_{1,9} = 4$, $R^2 = 0.28$, $p = 0.009$

Supplementary Table 11: Comparisons of Bray-Curtis dissimilarity values for bulk soil versus recolonized soil. Comparisons are between bacterial and fungal compositions at each time point. Overall comparison is a Kruskal-Wallis test followed by a Dunnett's post-hoc test.

Location	Overall	Individual timepoint comparison
Farm	Degrees of freedom = 3<br>H = 119,<br>$p < 0.001$	1 week: Z = 10, $q < 0.001$<br>10 weeks: Z = 4, $q < 0.001$
Forest	Degrees of freedom = 3<br>H = 123,<br>$p < 0.001$	1 week: Z = 7, $q < 0.001$<br>10 weeks: Z = 3, $q = 0.01$
Grass	Degrees of freedom = 3<br>H = 250,<br>$p < 0.001$	1 week: Z = 15, $q < 0.001$<br>10 weeks: Z = 6, $q < 0.001$
CCC	Degrees of freedom = 1<br>H = 15,<br>$p < 0.001$	NA*

*Only one time-point was sample

Supplementary Table 12: Core bacterial genera present in 100 % of samples and more than 0. 1 % averaged relative abundance at the 1-week timepoint.

Farm	Grass	Forest	All locations
Paenarthrobacter<br>Chryseobacterium<br>Achromobacter<br>Ensifer<br>Unclassified Micrococcaceae<br>Sphingobacterium<br>Curtobacterium	Novosphingobium<br>Flavobacterium<br>Paenibacillus<br>Bdellovibrio<br>Unclassified Comamonadaceae<br>Brevundimonas<br>Caulobacter<br>Devosia<br>Luteibacter<br>Pseudomonas<br>Unclassified Oxalobacteraceae<br>Acinetobacter	Novosphingobium<br>Flavobacterium<br>Paenibacillus<br>Duganella<br>Unclassified Comamonadaceae<br>Brevundimonas<br>Cupriavidus<br>Devosia<br>Stenotrophomonas<br>Pseudomonas<br>Unclassified Oxalobacteraceae<br>Acinetobacter	Pedobacter<br>Dyadobacter<br>Allorhizobium-Neorhizobium-Pararhizobium-Rhizobium<br>Sphingomonas<br>Unclassified Enterobacteriaceae<br>Stenotrophomonas<br>Massilia

Supplementary Table 13: Core fungal genera present in 100 % of samples and more than 0. 1 % averaged relative abundance at the 1-week timepoint.

Farm	Grass	Forest	All locations
Fusarium	Cryptococcus<br>Unclassified Fungi<br>Fusarium<br>Vishniacozyma<br>Pyrenochaetopsis<br>Hannaella<br>Unclassified Phaeosphaeriaceae<br>Papiliotrema<br>Cladosporium<br>Saitozyma	Alternaria<br>Cladosporium	None

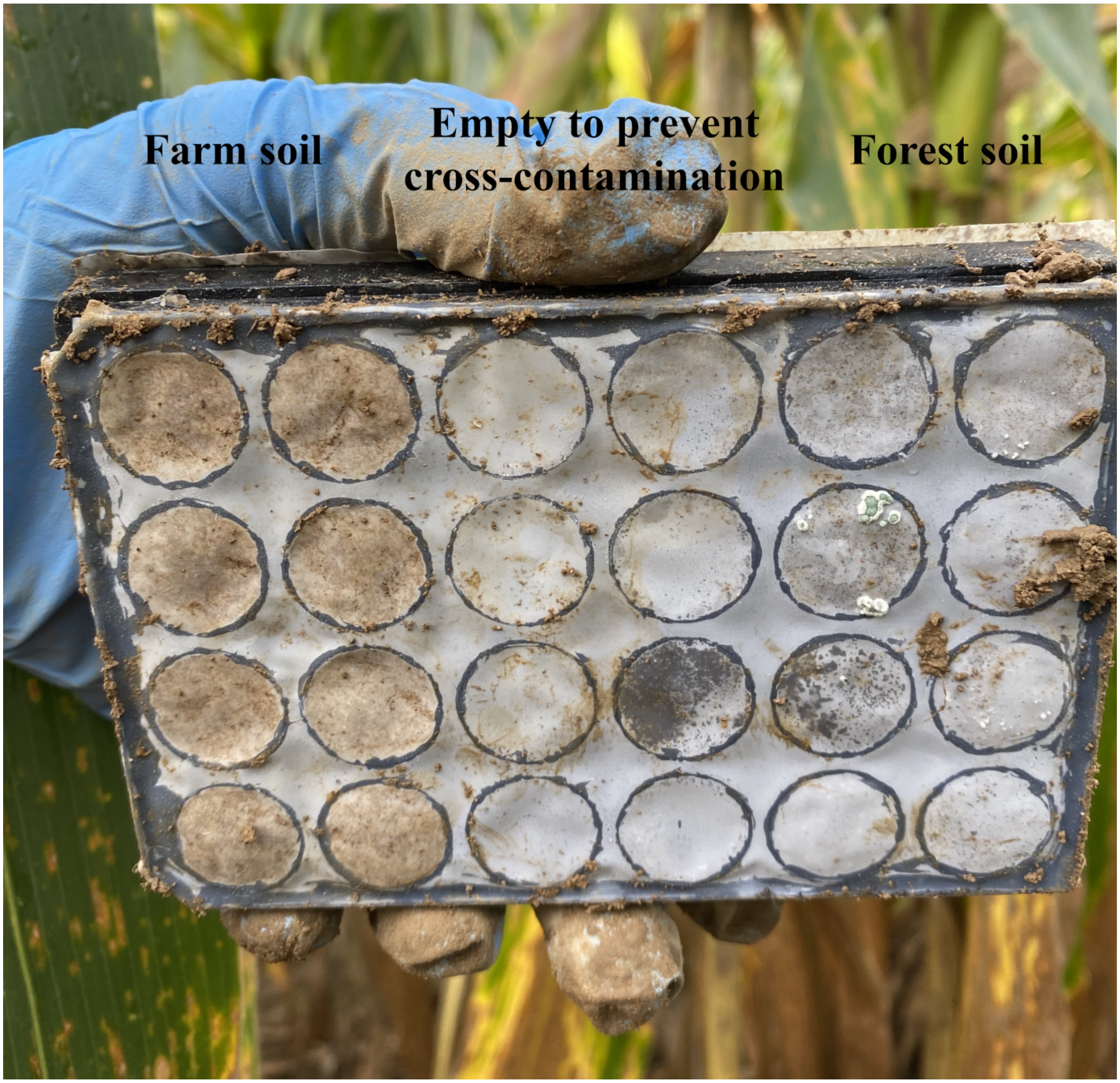

Supplementary Figure 1: A constructed microbial trap with an 18 μm nylon membrane. Microbial trap had been deployed into an agricultural corn field.

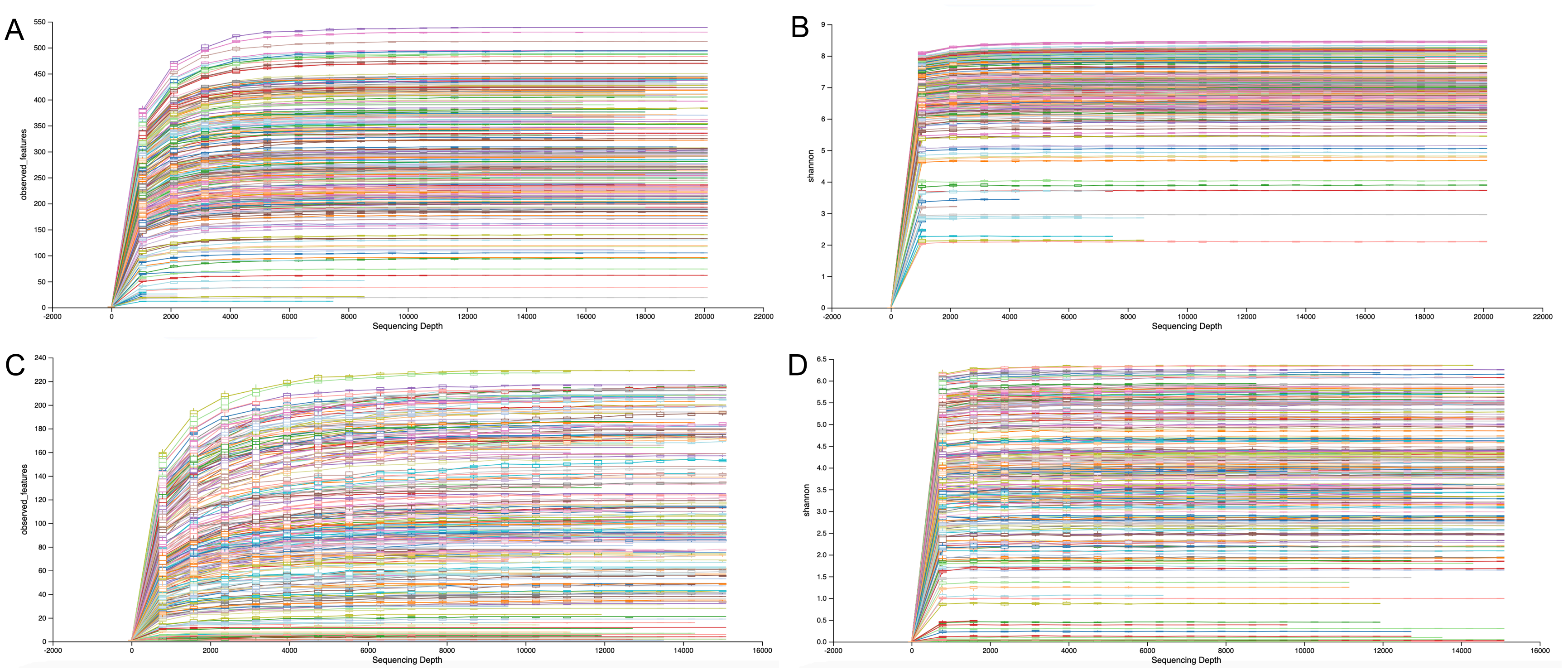

Supplementary Figure 2: Rarefaction curves. A and B are bacterial species richness and diversity, respectively. C and D are fungal species richness and diversity, respectively.

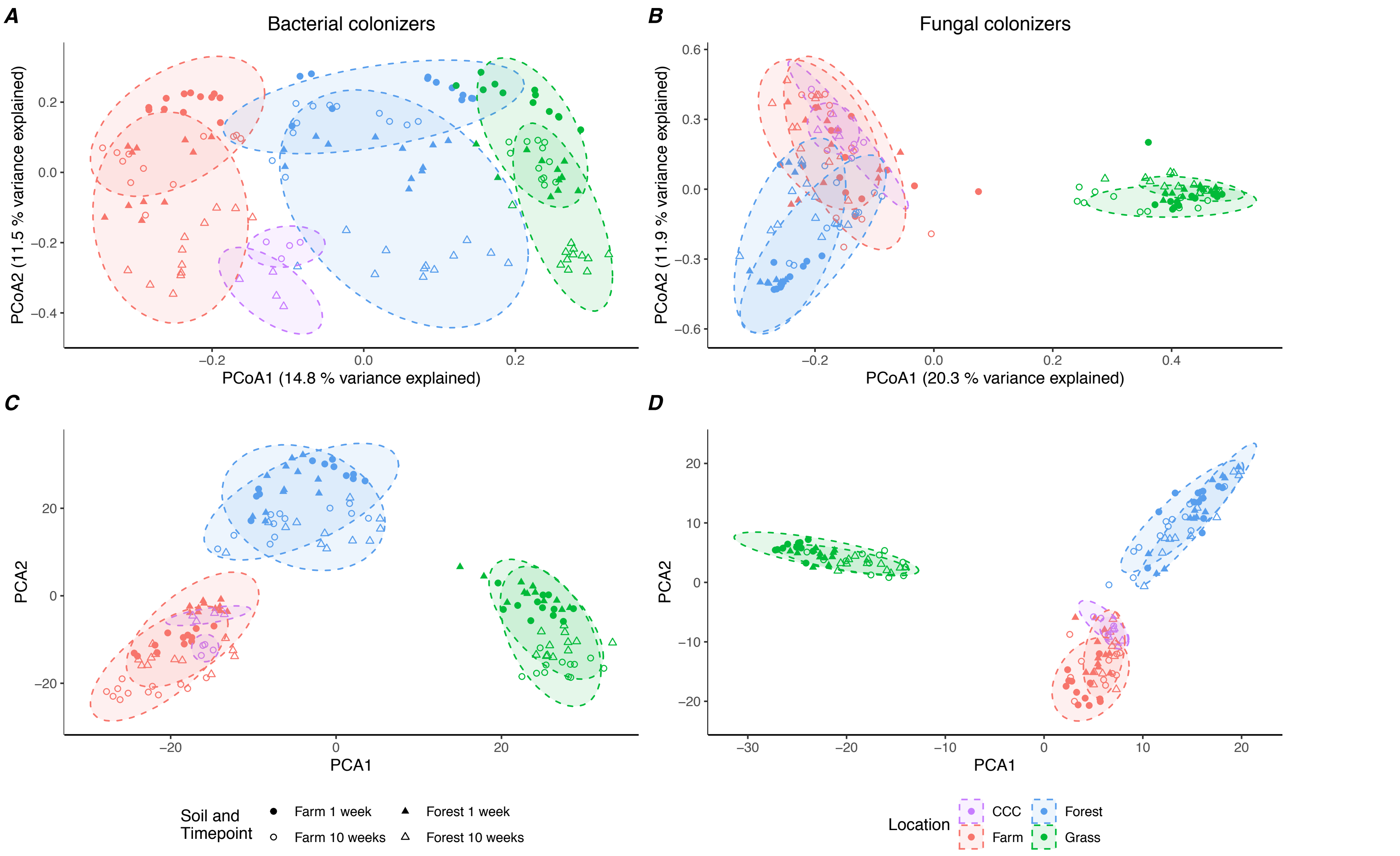

Supplementary Figure 3: PCoA and PCA ordinations of active microbial colonizer and local bulk soil microbial composition. Panels A and B are PCoA ordinations made from rarefied data and a Bray-Curtis index. Panels C and D are PCA ordinations made from unrarefied center-log-transformed data and a Euclidean distance

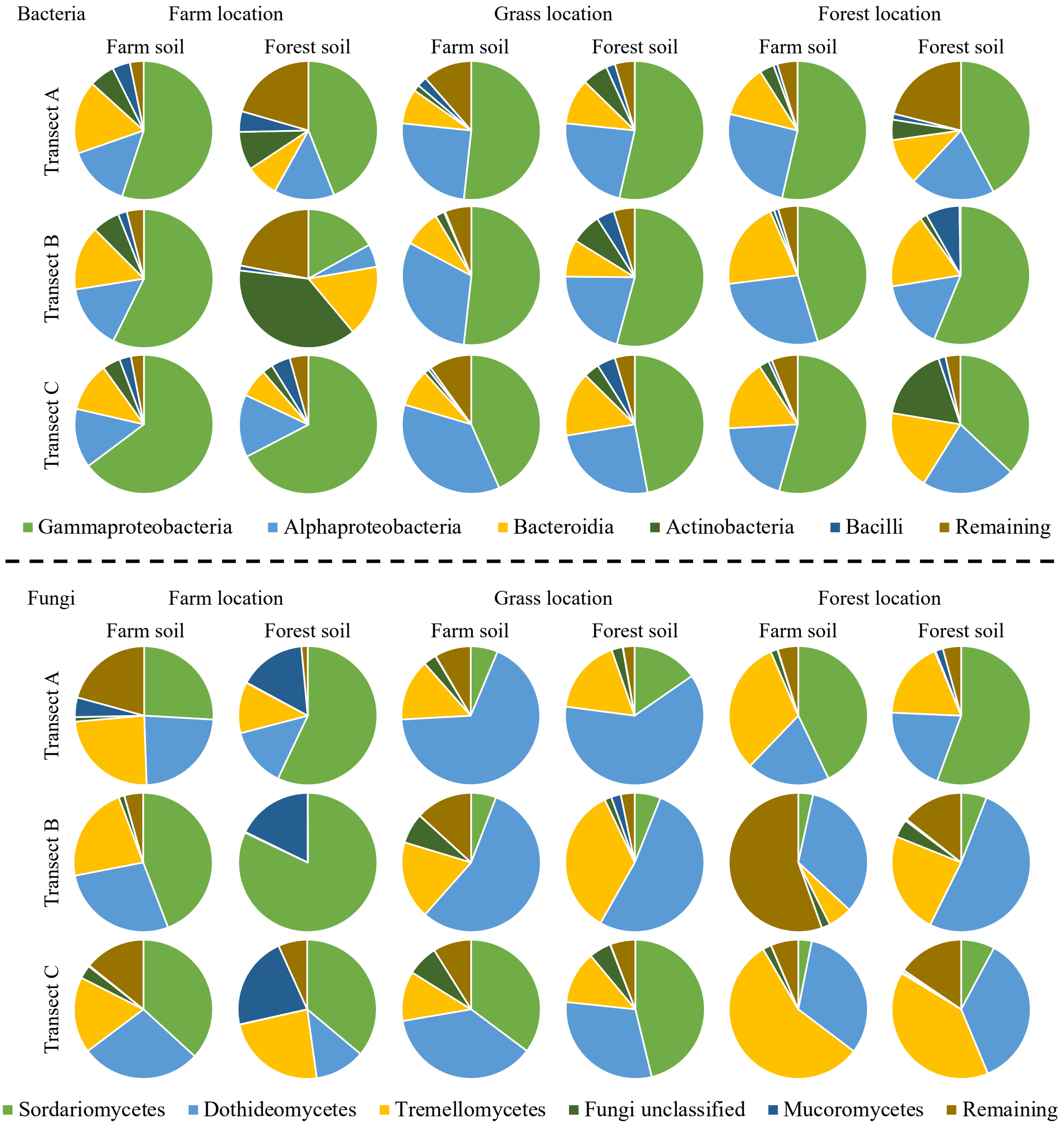

Supplementary Figure 4: Compositional plots for bacterial and fungal compositions at the 1-week collection timepoint. Data were summarized at the Class level and averaged across replicates within a transect per location. Transect A, B and C were the replicated transects within a location. The five most abundant taxa are shown.

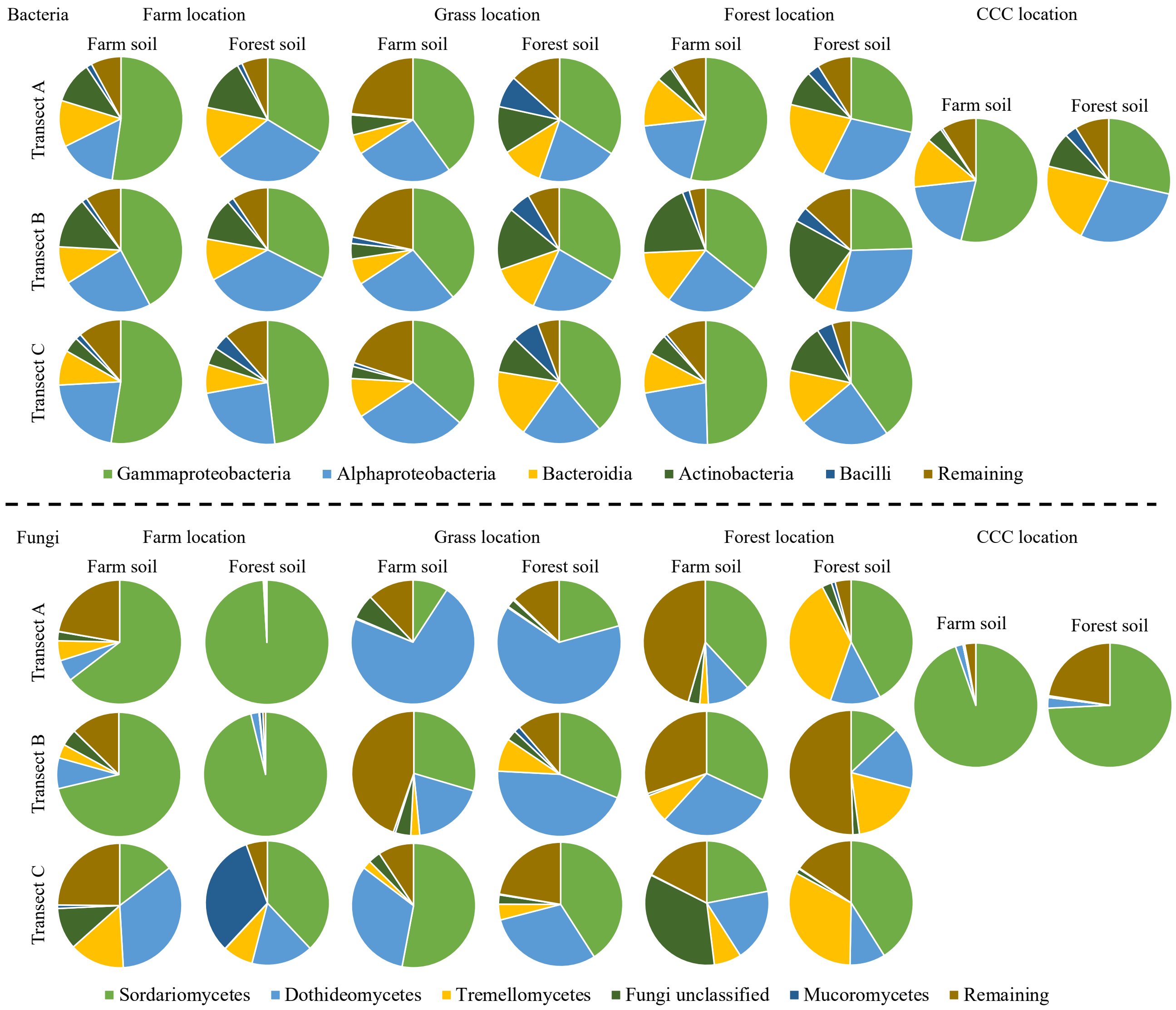

Supplementary Figure 5: Compositional plots for bacterial and fungal compositions at the 10-week collection timepoint. Data were summarized at the Class level and averaged across replicates within a transect per location. Transect A, B and C were the replicated transects within a location. The five most abundant taxa are shown.

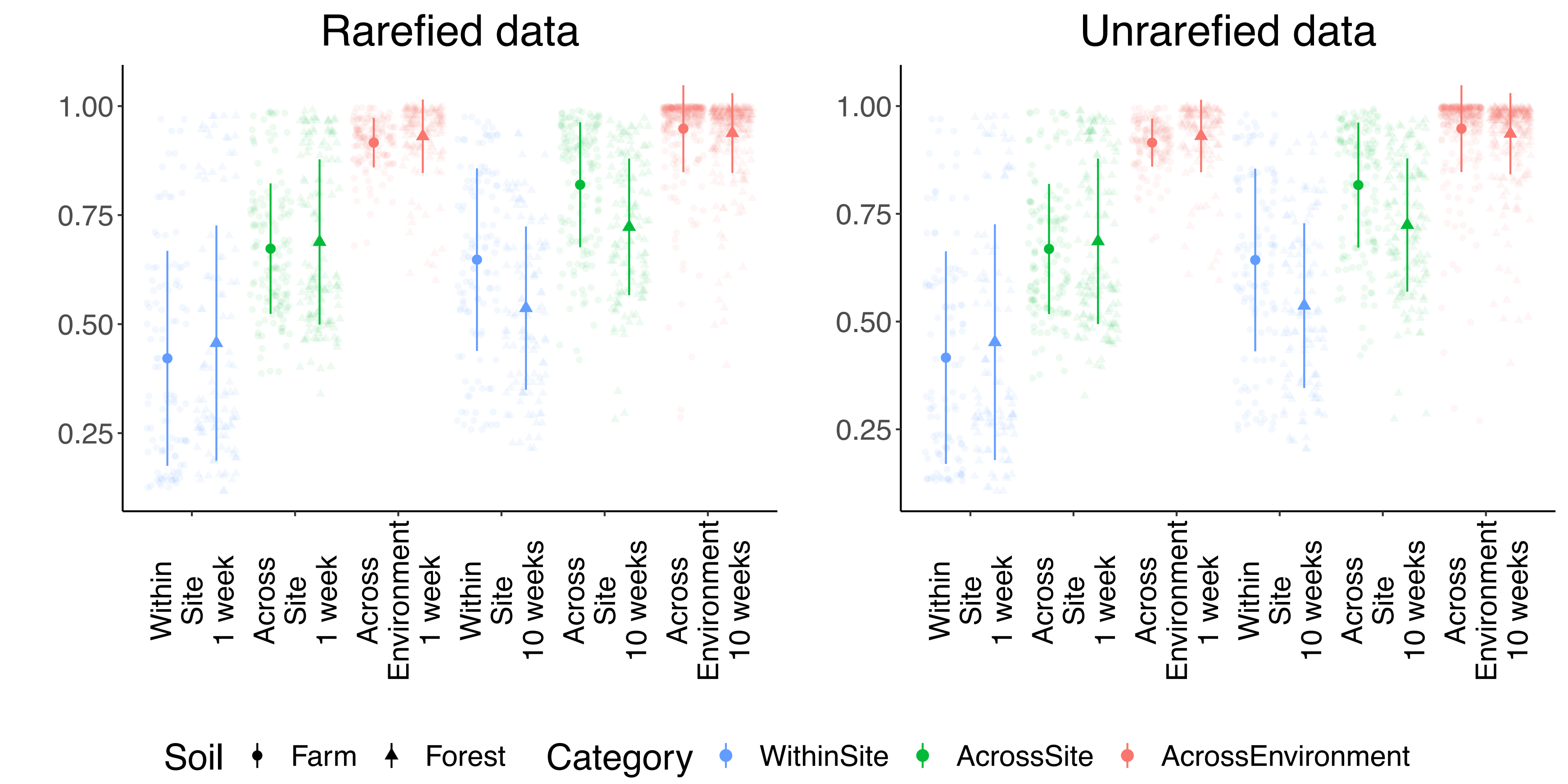

Supplementary Figure 6: Bray-Curtis dissimilarities of fungal compositions within-site, across-sites and across-locations. Showed data is for rarefied and unrarefied fungal composition data. Negligible differences were observed between the two datasets.

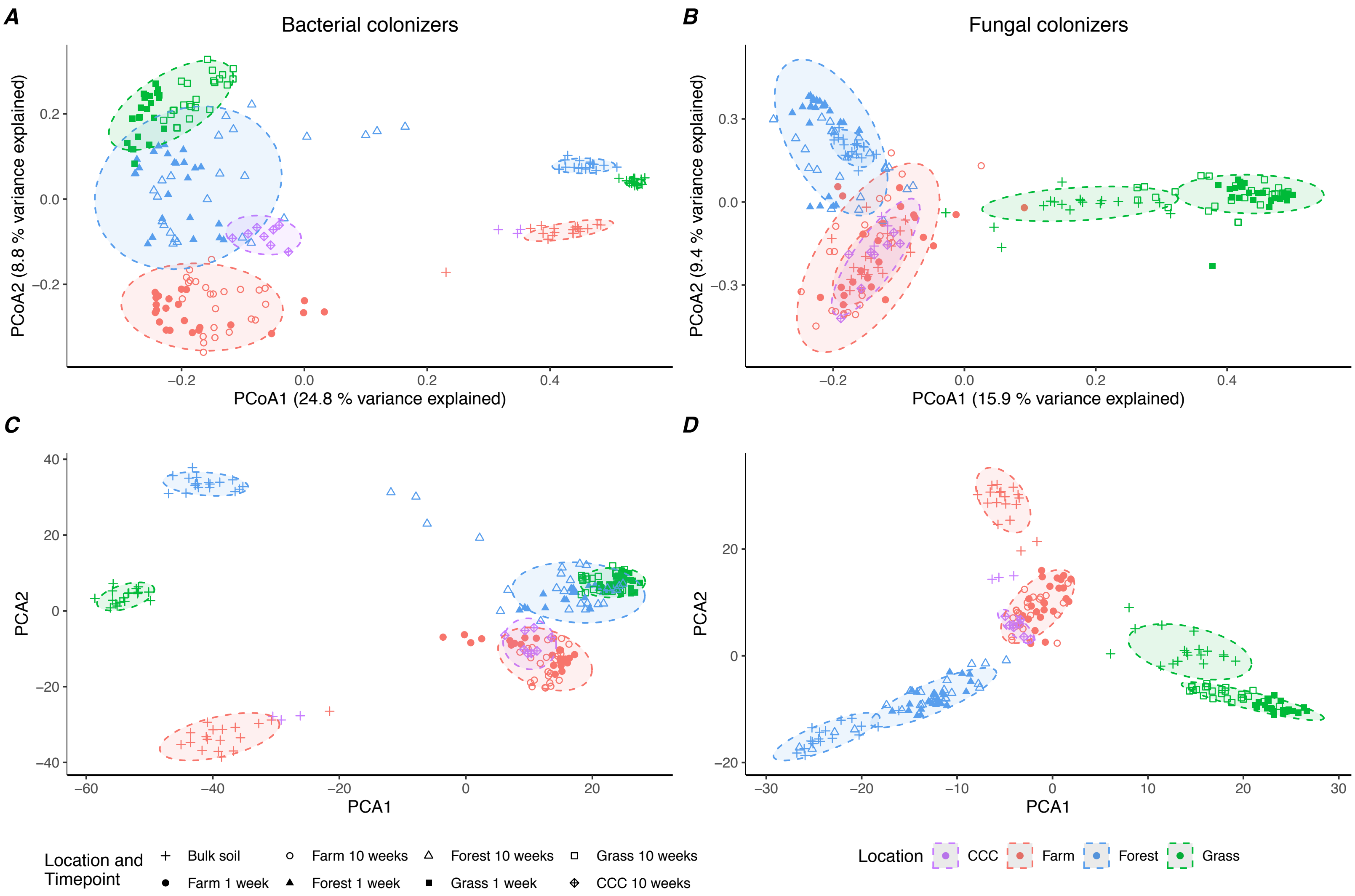

Supplementary Figure 7: PCoA and PCA ordinations of active microbial colonizer and local bulk soil microbial composition. Panels A and B are PCoA ordinations made from rarefied data and a Bray-Curtis index. Panels C and D are PCA ordinations made from unrarefied center-log-transformed data and a Euclidean distance.
